# Long-term Neurological Consequences of COVID-19 in Patients With Pre-existing Alzheimer’s and Parkinson’s Disease: A Comprehensive Review

**DOI:** 10.1177/26331055251342755

**Published:** 2025-05-29

**Authors:** Kelechi Wisdom Elechi, Ogundipe Oyepeju Nkem, Ndubuisi Timothy Chibueze, Ubalaeze Solomon Elechi, Kenechukwu Franklin Chimaobi

**Affiliations:** 1 Integrated Biomedical Sciences, University of Texas Health San Antonio, San Antonio, TX, USA; 2 Stephen M. Ross School of Business, University of Michigan, Ann Arbor, MI, USA; 3 Department of Microbiology, Nnamdi Azikiwe University, Awka, Anambra State, Nigeria; 4 Department of Radiography and Radiological Sciences, Faculty of Health Science and Technology, University of Nigeria Enugu Campus, Enugu, Nigeria; 5 Drug Delivery and Nanomedicines Research Laboratory, Department of Pharmaceutics, Faculty of Pharmaceutical Sciences, University of Nigeria, Nsukka, Enugu State, Nigeria

**Keywords:** COVID-19, Alzheimer’s disease, Parkinson’s disease, neurological consequences, neurodegeneration

## Abstract

SARS-CoV-2, the causative agent of COVID-19, has profound systemic effects, including significant impacts on the central nervous system (CNS). Emerging evidence suggests a potential link between SARS-CoV-2-induced neuroinflammation and the exacerbation or initiation of neurodegenerative diseases such as Alzheimer’s disease (AD) and Parkinson’s disease (PD). This review explores the mechanisms by which SARS-CoV-2 may contribute to neurodegenerative processes. We first discuss the pathways of viral entry into the CNS, including transneuronal and hematogenous routes, leading to blood-brain barrier (BBB) dysfunction. Neuroinflammation, mediated by the activation of microglia and astrocytes and the release of pro-inflammatory cytokines such as IL-6, TNF-α, and IL-1β, is highlighted as a critical factor exacerbating neuronal damage. Oxidative stress and vascular damage are further examined as complementary mechanisms promoting neurodegeneration. In addition, we review how SARS-CoV-2 infection influences proteinopathies by accelerating the aggregation of pathological proteins like alpha-synuclein, tau, and TDP-43, contributing to disease progression in PD, AD, and related disorders. Clinical studies reporting cognitive and motor dysfunctions in post-COVID-19 patients with pre-existing neurodegenerative diseases are also summarized. Finally, this review identifies knowledge gaps and emphasizes the need for further research to clarify the long-term neurological consequences of SARS-CoV-2 infection. Understanding these mechanisms is critical for developing targeted therapeutic strategies to mitigate the risk of neurodegeneration in vulnerable populations.

## Background

The severe acute respiratory syndrome coronavirus 2 (SARS-CoV-2), responsible for the COVID-19 pandemic, which has evolved in different waves worldwide,^1-3^ has infected millions of people worldwide, causing significant morbidity and mortality. While initially recognized primarily as a respiratory illness, it has become increasingly evident that COVID-19 can have profound and lasting neurological implications.^4^ The neurotropic properties of SARS-CoV-2 have been well-documented, with the virus capable of affecting the central nervous system (CNS) through various mechanisms.^5^ Emerging evidence indicates that SARS-CoV-2 infection can lead to neuroinflammation, which may exacerbate conditions like Alzheimer’s and Parkinson’s disease.^6^ Patients with pre-existing neurodegenerative conditions, particularly Alzheimer’s disease (AD) and Parkinson’s disease (PD), represent a uniquely vulnerable population in the context of the COVID-19 pandemic. These individuals not only face an increased risk of severe COVID-19 but may also experience long-term exacerbation of their underlying neurological condition.^7^ The intersection of COVID-19 and neurodegenerative diseases presents a complex challenge for clinicians and researchers alike, necessitating a comprehensive understanding of their interactions and long-term consequences.

This review aims to synthesize current research on the long-term neurological consequences of COVID-19 in patients with pre-existing AD and PD. We will examine the potential mechanisms by which SARS-CoV-2 infection may exacerbate or accelerate neurodegenerative processes, review recent clinical findings, discuss the challenges in patient management, and explore future research directions. By comprehensively analyzing the available evidence, we hope to provide valuable insights for clinicians managing these complex cases and to identify critical areas for future investigation.

## Pathophysiological Mechanisms

**Figure 1.**
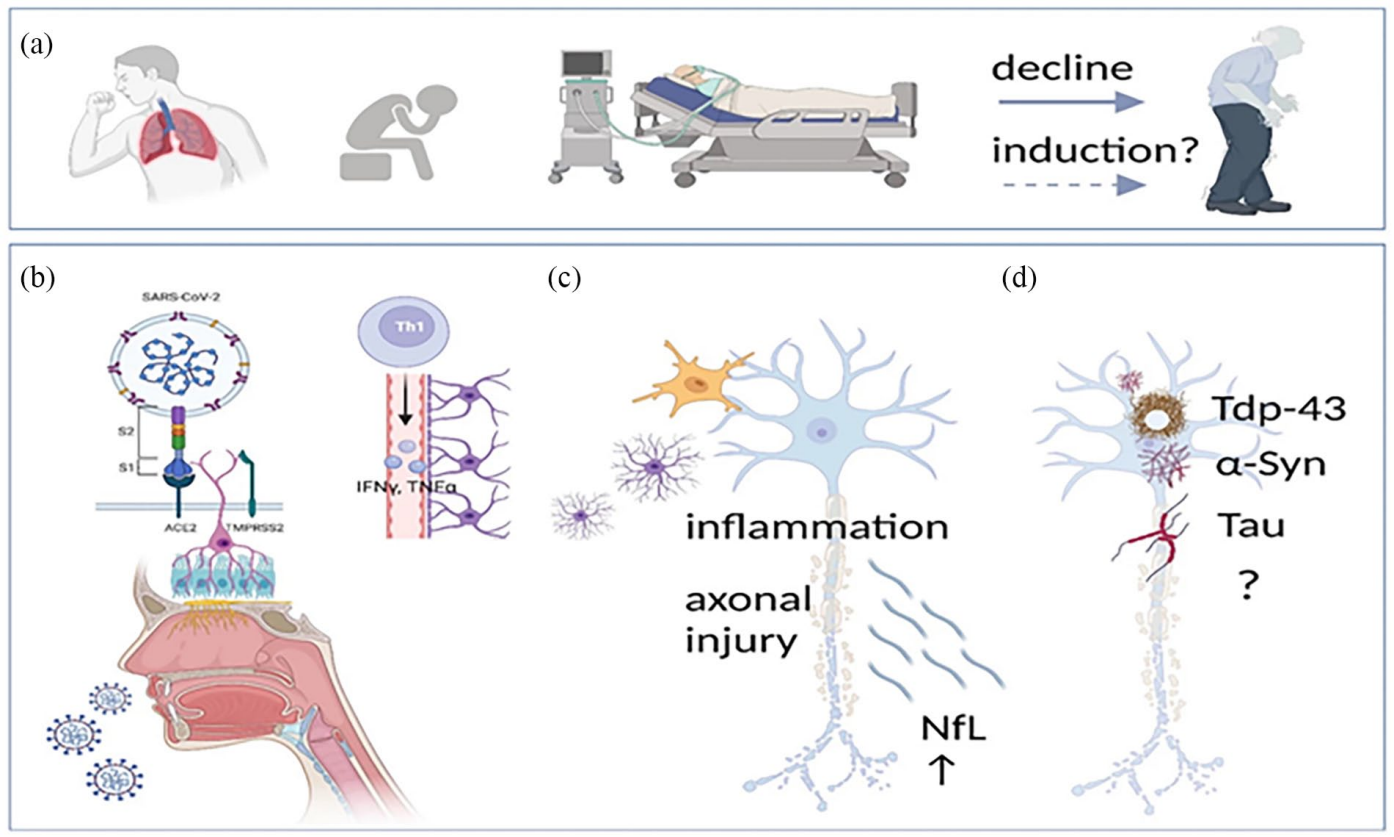
Impact of SARS-CoV-2 on neurodegenerative disease pathways. This figure illustrates the potential effects of SARS-CoV-2 infection on neurodegenerative disease mechanisms: (a) Clinical presentations include neurological symptoms, severe sepsis, and potential exacerbation or induction of neurodegenerative disorders, (b) SARS-CoV-2 enters the brain via nasal and oral mucosa, leading to neuroinflammation and blood-brain barrier (BBB) dysfunction, (c) Activation of microglia, astrocytes, and pro-inflammatory cytokines results in neuronal damage and the release of neurofilament light chain (NfL), and (d) Neuronal damage by SARS-CoV-2 may contribute to proteinopathies such as Parkinson’s disease (PD), Alzheimer’s disease (AD), and amyotrophic lateral sclerosis (ALS). Reproduced with permission under the Creative Commons Attribution License (CC BY) from Lingor et al.^9^ Published by Journal of Neural Transmission. Citation: Lingor et al., Journal of Neural Transmission, 129, 1155–1167 (2022). https://doi.org/10.1007/s00702-022-02500-w.

Understanding the pathophysiological mechanisms underlying the interaction between COVID-19 and neurodegenerative diseases is crucial for developing effective management strategies and potential interventions. Several key mechanisms have been proposed and investigated. However, it is crucial to differentiate between neurological symptoms directly caused by SARS-CoV-2 invasion of the CNS and those arising from systemic inflammatory responses, which this review partly does. Empirical studies have demonstrated that SARS-CoV-2 can directly infect neuronal cells, leading to cell death.^8^ SARS-CoV-2 interacts with multiple neurological pathways, potentially exacerbating neurodegenerative processes through mechanisms such as neuroinflammation, blood-brain barrier (BBB) dysfunction, and oxidative stress. These interactions are illustrated in Figure 1, which outlines the clinical presentation of SARS-CoV-2 infection, its cellular and molecular mechanisms, and its potential contribution to proteinopathies like Parkinson’s disease (PD), Alzheimer’s disease (AD), and amyotrophic lateral sclerosis (ALS)

### Neuroinflammation

**Figure 2.**
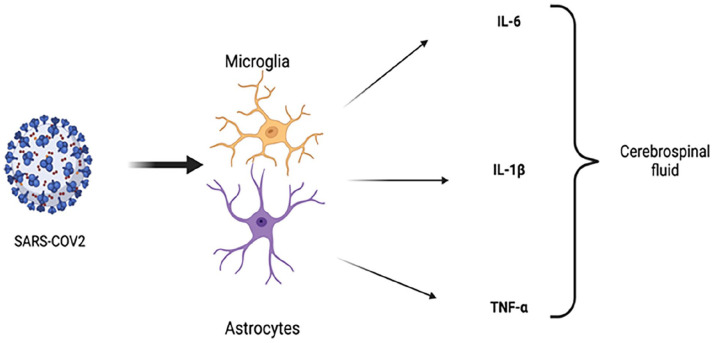
Activation of microglia and astrocytes in neuroinflammation. This figure depicts the activation of microglia and astrocytes triggered by SARS-CoV-2 infection, leading to the release of pro-inflammatory cytokines such as IL-6, TNF-α, and IL-1β. These processes exacerbate the neuroinflammatory environment, contributing to neuronal damage and accelerated neurodegeneration. (Created with https://biorender.com/)

Chronic neuroinflammation is a hallmark of both AD and PD, playing a crucial role in disease progression.^10^ SARS-CoV-2 infection can trigger a robust inflammatory response, potentially exacerbating this pre-existing neuroinflammation.^11-13^ The virus has been shown to activate microglia and astrocytes, leading to the release of pro-inflammatory cytokines and chemokines.^14-16^ In addition, Brain-targeted SARS-CoV-2 infection can induce microglia activation, leading to chronic inflammation and eventual neurodegeneration.^17^ A study by Nuber-Chapier et al. demonstrated elevated levels of pro-inflammatory cytokines, including IL-6, TNF-α, and IL-1β, in the cerebrospinal fluid (CSF) of AD patients following COVID-19, persisting for up to 6 months post-infection.^18^ Similarly, Mysiris et al. found increased levels of neuroinflammatory markers in the CSF of PD patients who had recovered from COVID-19, correlating with worsened motor symptoms.^19^ The sustained neuroinflammatory response following COVID-19 may create a neurotoxic environment, potentially accelerating neurodegeneration.^20^ This is particularly concerning in the context of AD and PD, where the brain’s inflammatory state is already heightened. Table 1 distills the principal cytokines and transcription factors—IL‑6, TNF‑α, IL‑1β and NF‑κB—implicated in COVID‑19‑amplified neuroinflammation and outlines their specific repercussions for Alzheimer’s and Parkinson’s pathology. In addition, as illustrated in Figure 2, SARS‑CoV‑2–induced activation of microglia and astrocytes unleashes a cascade of IL‑6, TNF‑α and IL‑1β that sustains a neurotoxic inflammatory milieu.

**Table 1.** Key Neuroinflammatory Markers.

Marker	Role in neuroinflammation	Implications for AD/PD Post-COVID-19
IL-6	Pro-inflammatory cytokine; promotes neuroinflammation	Elevated in cerebrospinal fluid; linked to worsened symptoms^13^
TNF-α	Triggers apoptosis and inflammation	Associated with accelerated neurodegeneration^13^
IL-1β	Induces fever and inflammation; activates glial cells	Persistent elevation post-COVID-19; worsens cognitive decline^14^
NF-κB	Key regulator of inflammatory response	Increased activation in COVID-19; exacerbates neuronal damage^16^

### Oxidative Stress

Oxidative stress plays a crucial role in the pathogenesis of neurodegenerative diseases, contributing to mitochondrial dysfunction, protein aggregation, and neuronal death.^21,22^ SARS-CoV-2 exacerbates neuro-inflammation, increases oxidative stress, and consequently contributes to neuronal cell death.^23^ COVID-19 has been associated with increased systemic oxidative stress, which may further compromise already vulnerable neuronal populations in AD and PD patients.^24,25^ Lee et al. found elevated markers of oxidative stress, including malondialdehyde and 8-hydroxy-2'-deoxyguanosine, in the plasma of PD patients who had recovered from COVID-19.^26,27^ These elevations correlated with worsened motor symptoms and accelerated disease progression.^28^ In AD patients, Reiken et al. reported increased levels of oxidized proteins and lipids in post-mortem brain tissue of individuals who had contracted COVID-19, compared to AD patients without a history of infection.^29^ The additional oxidative burden imposed by COVID-19 may overwhelm the already compromised antioxidant defenses in neurodegenerative conditions, potentially accelerating cellular damage and disease progression.^30^

### Vascular Damage and Blood-Brain Barrier Dysfunction

COVID-19 can cause significant endothelial dysfunction and microthrombi formation, potentially worsening cerebrovascular aspects of neurodegenerative diseases.^31,32^ SARS-CoV-2 infection has been shown to disrupt BBB integrity, a phenomenon also observed with other viral infections, potentially leading to neuroinflammation and neuronal damage.^33^ This is particularly relevant for AD, where vascular pathology often coexists with typical AD pathology, contributing to cognitive decline.^34^ A study by Greene et al used advanced neuroimaging techniques to demonstrate increased blood-brain barrier (BBB) permeability in AD patients following COVID-19 infection, persisting for several months post-recovery.^35^ Studies have identified ACE2 receptors on human brain microvascular endothelial cells, suggesting that SARS-CoV-2 may enter the CNS via hematogenous routes, potentially disrupting the blood-brain barrier.^36,37^ This increased BBB permeability may allow for greater infiltration of peripheral immune cells and inflammatory mediators into the CNS, further exacerbating neuroinflammation and neurodegeneration.^38^ In PD, where cerebrovascular health is increasingly recognized as a contributing factor to disease progression, COVID-19-induced vascular damage may have significant long-term consequences. Huang al. reported accelerated white matter hyperintensity progression in PD patients with a history of COVID-19, suggesting ongoing vascular injury.^39,40^

### Direct Viral Effects

While controversial, some studies suggest that SARS-CoV-2 can directly infect neurons or glial cells.^41,42^ The virus utilizes the angiotensin-converting enzyme 2 (ACE2) receptor for cell entry, which is expressed in various brain regions, including those affected in AD and PD.^43-45^ Liu et al demonstrated SARS-CoV-2 RNA in post-mortem brain tissue of AD patients who died from COVID-19, particularly in regions already affected by AD pathology, such as the hippocampus and cortex.^17,46^ This suggests that these already vulnerable brain regions may be particularly susceptible to direct viral invasion. In PD, there is growing concern that SARS-CoV-2 may directly impact dopaminergic neurons. A study by Han et al. found evidence of SARS-CoV-2 proteins in dopaminergic neurons of the substantia nigra in post-mortem tissue from PD patients with COVID-19 history.^47^ While the long-term implications of this finding are not yet clear, it raises the possibility of direct viral contribution to dopaminergic neuron loss in PD.

### Protein Aggregation and Clearance

Both AD and PD are characterized by the accumulation of specific protein aggregates – amyloid-β and tau in AD, and α-synuclein in PD. Emerging evidence suggests that COVID-19 may influence these protein aggregation processes and impair cellular clearance mechanisms.^48,49^ Duff et al. used plasma biomarkers to assess changes related to Alzheimer’s disease pathology before and after serology-confirmed SARS-CoV-2 infections in the UK Biobank. They found that SARS-CoV-2 infection was associated with biomarkers indicating increased β-amyloid pathology, including reduced plasma Aβ42:Aβ40 ratio and, in more vulnerable participants, lower plasma Aβ42 and higher plasma pTau-181. These biomarker changes were more pronounced in participants who had been hospitalized with COVID-19 or had previously reported hypertension and were linked to brain structural imaging patterns associated with Alzheimer’s disease, lower cognitive test scores, and poorer overall health evaluations.^50^ The mechanisms behind this acceleration are not fully understood but may involve increased protein production, impaired clearance, or both. In PD, Wang et al. reported increased α-synuclein aggregation in cellular models exposed to SARS-CoV-2 proteins, particularly the spike protein.^51^ While this finding needs to be confirmed in human studies, it suggests a potential direct link between SARS-CoV-2 infection and accelerated α-synuclein pathology. Furthermore, COVID-19 has been shown to impair autophagy, a crucial cellular process for clearing protein aggregates and damaged organelles.^52^ This impairment may contribute to the accelerated accumulation of pathological proteins in both AD and PD. Table 2 presents an integrated synopsis of the key mechanisms—neuroinflammation, oxidative stress, vascular injury, direct viral effects and protein aggregation—through which SARS‑CoV‑2 infection accelerates neurodegenerative processes.

**Table 2.** Pathophysiological Mechanisms Linking COVID-19 to Neurodegenerative Processes.

Mechanism	Role	Implications for AD/PD Post-COVID-19	References
Neuroinflammation	Activation of microglia and astrocytes, release of pro-inflammatory cytokines (IL-6, TNF-α)	Exacerbates pre-existing neuroinflammatory states, accelerating neurodegeneration	Ref.^5-7^
Oxidative stress	Increased ROS production, mitochondrial dysfunction	Aggravates protein aggregation and neuronal loss in AD and PD	Liu et al^17^, Nuber-Champier et al^18^
Vascular damage and BBB dysfunction	Endothelial damage, microthrombi, increased BBB permeability	Facilitates inflammatory cell infiltration, worsening neuronal damage	Ref.^24-26^
Direct viral effects	Potential neuronal infection via ACE2 receptors	May cause direct damage to dopaminergic and hippocampal neurons	Pelle et al^31^, Varga et al^32^
Protein aggregation	Impaired clearance of Aβ, tau, and α-synuclein	Accelerates hallmark pathologies of AD (Aβ, tau) and PD (α-synuclein)	Zou et al^40^, Beckman et al^41^

## Clinical Manifestations and Long-term Consequences of Alzheimer’s Disease

The long-term neurological consequences of COVID-19 in patients with pre-existing AD are becoming increasingly apparent as more longitudinal studies are conducted. These consequences span cognitive, functional, and neuropsychiatric domains, potentially accelerating the overall disease course.

### Cognitive Decline

Multiple studies have reported accelerated cognitive decline in AD patients following COVID-19 infection. A study by Merla et al. found that COVID-19 had a significant impact on cognitive decline in elderly patients with dementia, with those who contracted COVID-19 being 3.5 times more likely to experience cognitive deterioration.^53^ Additionally, the rate of cognitive decline, as measured by the Mini-Mental State Examination (MMSE), was found to accelerate in COVID-19 patients, with their MMSE scores dropping by 3.3 points per year, compared to 1.7 points in those who did not have the disease. The study also revealed that COVID-19 patients had a higher incidence of new institutionalization, highlighting the detrimental effects of the virus on cognitive function and daily living activities in this vulnerable population.

Similarly, a study by Delgado-Alonso et al. used a comprehensive neuropsychological battery to determine the characteristics of cognitive dysfunction in patients reporting cognitive complaints after COVID-19.^54^ The study found that patients who reported cognitive complaints following COVID-19 exhibited diminished performance in several cognitive domains, particularly in attention and executive function, as well as episodic memory and visuospatial processing. These deficits were primarily observed in processing speed, divided and selective attention, working memory, and inhibition. The cognitive dysfunction was notably correlated with olfactory dysfunction, with smaller correlations to sleep quality and anxiety, but not depression. The findings suggest that cognitive impairment post-COVID-19 is particularly pronounced in attention and executive functioning.

The mechanisms underlying this accelerated cognitive decline are likely multifactorial, involving the interplay of neuroinflammation, oxidative stress, and potentially direct viral effects on vulnerable brain regions. The hippocampus, crucial for memory function and already a primary target in AD, may be particularly susceptible to COVID-19-related damage.^55^

### Neuropsychiatric Symptoms

COVID-19 infection in AD patients has been associated with the worsening of existing neuropsychiatric symptoms and the emergence of new ones. A study by Cinar et al. found that Alzheimer’s disease (AD) patients experienced a significant worsening of neuropsychiatric symptoms during the COVID-19-related total lockdown compared to the partial lockdown.^56^ The mean Neuropsychiatric Inventory (NPI) score was significantly higher during total lockdown (22.9) than partial lockdown (17.7). Factors such as the presence of comorbidities, reduced mobility, and limited social interactions contributed to the worsening of neuropsychiatric symptoms during total lockdown. Regression analysis showed that the Clinical Dementia Rating (CDR) score was the most influential factor affecting the neuropsychiatric status of AD patients during both lockdown periods, highlighting the impact of severe restrictions on their mental health. The exacerbation of neuropsychiatric symptoms may be related to the neuroinflammatory and neurovascular effects of COVID-19, as well as the psychological impact of the infection and associated isolation.^57,58^

## Clinical Manifestations and Long-term Consequences in Parkinson’s Disease

**Figure 3.**
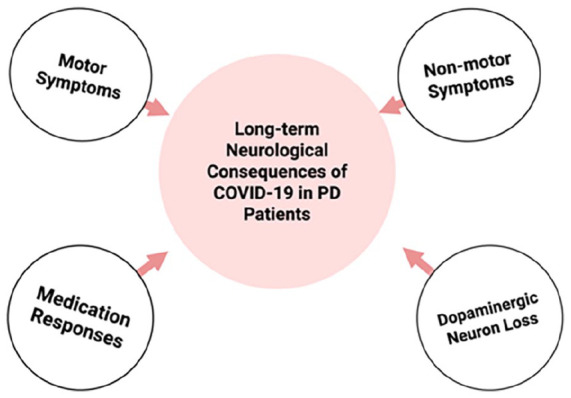
Long-term neurological consequences of COVID-19 in Parkinson’s disease patients.

The long-term neurological consequences of COVID-19 in patients with pre-existing PD are equally concerning, affecting both motor and non-motor aspects of the disease. Figure 3 synthesizes the spectrum of post‑COVID‑19 motor and non‑motor sequelae now documented in Parkinson’s disease patients.

### Motor Symptoms

Several studies have reported worsening of motor symptoms in PD patients following COVID-19 infection. A study by Wang et al. found that patients with a history of COVID-19 infection had a significantly increased risk of developing Parkinson’s disease within the first year after infection.^59^ The risk was highest at 6 months post-infection, with elevated odds of new-onset Parkinson’s disease at 3, 6, 9, and 12 months compared to those without a COVID-19 infection. However, after twelve months, the risk difference between the COVID-19 and non-COVID-19 groups became non-significant. This suggests that COVID-19 may transiently increase the risk of Parkinson’s disease within the first year following infection.

Tremor, rigidity, and bradykinesia have all been reported to worsen following COVID-19 infection in PD patients. A study by Xu et al found that COVID-19 manifestations in people with Parkinson’s disease (PD) were heterogeneous, similar to the general population, ranging from asymptomatic cases to death. Common symptoms included fever/chills, fatigue, cough, weight loss, and muscle pain.^60^ Additionally, many PD patients experienced worsening or new onset of both motor symptoms (such as dyskinesia, rigidity, and balance disturbances) and non-motor symptoms (such as anxiety, depression, and insomnia) during their COVID-19 illness. The study did not find enough evidence to suggest that PD is an independent risk factor for severe COVID-19 or death, but larger controlled studies are needed to confirm this.

The mechanisms underlying this acceleration of motor symptoms are likely multifactorial, involving increased neuroinflammation, oxidative stress, and potentially direct viral effects on dopaminergic neurons.^61^

### Non-motor Symptoms

COVID-19 infection has also been associated with exacerbation of non-motor symptoms in PD. Several studies have reported newly emerging or acutely deteriorating non-motor symptoms in PD patients infected with SARS-CoV-2, with some of these symptoms persisting as part of the long-COVID syndrome.^62^ Additionally, the COVID-19 pandemic has negatively impacted the mental health and quality of life of PD patients due to disruptions in healthcare services and social restrictions.^24,63^

COVID-19 infection has been associated with exacerbation of cognitive impairment and autonomic dysfunction, two prominent non-motor features of Parkinson’s disease (PD). PD patients who recovered from COVID-19 demonstrated a more rapid decline in executive function and attention compared to those without a history of COVID-19 infection1. This accelerated cognitive deterioration may be attributed to a combination of factors, including the direct neurological impact of SARS-CoV-2, inflammatory responses, and the psychosocial stress of the pandemic.^62,64^ Additionally, autonomic dysfunction in PD patients has been observed to worsen following COVID-19 infection, with studies reporting increased severity of orthostatic hypotension and gastrointestinal symptoms.^65^ These findings highlight the potential long-term neurological consequences of COVID-19 in PD patients and underscore the need for close monitoring and targeted interventions to manage these exacerbated non-motor symptoms.

### Dopaminergic Neuron Loss

Recent research has raised concerns about the potential impact of COVID-19 on dopaminergic neuron loss in Parkinson’s disease (PD) patients. A study by Yang et al. used dopamine transporter (DAT) imaging to assess dopaminergic neuron integrity in PD patients before and after COVID-19 infection and observed accelerated loss of dopaminergic signal in those who had recovered from COVID-19.^66^ This finding suggests that SARS-CoV-2 infection may directly affect these vulnerable neurons, potentially explaining the more rapid progression of motor symptoms observed in some PD patients following COVID-19. The study demonstrated that hPSC-derived dopamine (DA) neurons are susceptible to SARS-CoV-2 infection, triggering an inflammatory and cellular senescence response. Furthermore, reduced numbers of neuromelanin+ and tyrosine-hydroxylase (TH)+ DA neurons and fibers were observed in a cohort of severe COVID-19 patients.^66^ These findings highlight the need for careful, long-term monitoring of neurological problems in COVID-19 patients, particularly those with pre-existing PD.

### Medication Response

Studies have reported changes in medication response among Parkinson’s disease (PD) patients following COVID-19 infection. Some PD patients who recovered from COVID-19 experienced increased “off” time and more frequent and severe motor fluctuations, despite maintaining stable levodopa dosing.^67^ This suggests that COVID-19 may alter the pharmacodynamics of antiparkinsonian medications, potentially through changes in blood-brain barrier permeability or dopamine metabolism2. Additionally, PD patients with a history of COVID-19 infection were found to require higher doses of levodopa to achieve similar motor symptom control compared to those without COVID-19 history.^67^ The deterioration of motor symptoms in PD patients after COVID-19 infection may be explained by various factors, including stress, physical inactivity, pharmacodynamic effects, and marked changes in routine and social isolation.^65^

## Management Challenges and Strategies

The long-term neurological consequences of COVID-19 in patients with pre-existing Alzheimer’s disease (AD) and Parkinson’s disease (PD) present significant challenges in clinical management. Continuous monitoring is crucial, with the need for more frequent assessments using validated tools to detect subtle cognitive and motor changes. Early detection of any cognitive or motor decline can enable timely intervention, such as medication adjustments or initiating rehabilitative therapies.^68^

### Cognitive Rehabilitation for AD

Cognitive rehabilitation has shown potential in managing AD patients who experience accelerated decline following COVID-19. Intensive cognitive training has been demonstrated to improve cognitive function and activities of daily living in AD patients. Implementing personalized cognitive training programs may be critical for mitigating the rapid decline seen post-COVID-19 in this population.^69^

### Physical Therapy and Motor Rehabilitation for PD

For PD patients, early initiation or intensification of physical therapy and exercise programs may help slow the progression of motor symptoms exacerbated by COVID-19. Physical therapy interventions, such as balance training, strength exercises, and gait training, have been shown to be effective in maintaining motor function in PD, and this may be especially important for post-COVID-19 recovery.^70,71^

### Medication Management in PD and AD

Medication management post-COVID-19 requires careful attention, as altered responses to antiparkinsonian drugs have been observed in PD patients following infection.^72^ Frequent medication reviews and dosage adjustments may be necessary to maintain optimal symptom control in PD. For AD patients, earlier use of cholinesterase inhibitors or memantine might be considered to address cognitive decline, though further research is needed to assess their efficacy in the post-COVID-19 context.^73^

### Managing Neuropsychiatric Symptoms

The management of exacerbated neuropsychiatric symptoms, such as anxiety, depression, and sleep disturbances, remains a key challenge for both AD and PD patients post-COVID-19. Cognitive-behavioral therapy (CBT) has shown promise in addressing these symptoms, and when pharmacological treatment is necessary, careful monitoring for drug interactions is essential.^74^ Personalized, non-pharmacological interventions may be crucial for enhancing patient outcomes.

### Exploring Neuroprotective Strategies

Emerging research suggests that intensive anti-inflammatory therapies and high-dose antioxidant supplementation may offer neuroprotective benefits for AD and PD patients recovering from COVID-19.^75^ While preliminary studies show potential, larger-scale validation is needed before these approaches become part of standard treatment protocols.

### Caregiver Support and Telehealth

Caregiver education and support are essential due to the potential for accelerated disease progression and increased care needs post-COVID-19. Telehealth interventions, which have proven valuable during the pandemic, can continue to play a role in providing ongoing support and monitoring for caregivers, helping them manage the increasing complexity of care.^74^

## Future Research Directions

While significant progress has been made in understanding the long-term neurological consequences of COVID-19 in patients with pre-existing AD and PD, many questions remain unanswered. Future research should focus on several key areas:

**Long-term Longitudinal Studies:** Larger, multi-center studies with longer follow-up periods are needed to fully characterize the long-term trajectory of neurological consequences in AD and PD patients following COVID-19. These studies should incorporate comprehensive clinical assessments, neuroimaging, and biomarker analyses to provide a complete picture of disease progression.

**Mechanistic Studies:** Further investigation into the molecular mechanisms underlying the interaction between SARS-CoV-2 and neurodegenerative processes is crucial. In vitro and animal studies, as well as post-mortem human tissue analyses, can provide valuable insights into how COVID-19 may accelerate neurodegeneration at the cellular and molecular levels.

**Biomarker Development:** The development and validation of biomarkers specific to COVID-19-related neurological damage in the context of AD and PD could improve early detection and monitoring of long-term consequences. Elevated levels of phosphorylated tau protein in cerebrospinal fluid may serve as a biomarker for accelerated Alzheimer’s progression post-COVID. Other biomarkers may include novel neuroimaging techniques and blood-based biomarkers.

**Therapeutic Interventions:** Research into targeted therapeutic interventions to mitigate the long-term neurological impact of COVID-19 in AD and PD patients is urgently needed. This may include repurposing existing drugs with neuroprotective properties or developing novel therapies targeting the specific mechanisms of COVID-19-related neurological damage. In addition, cognitive rehabilitation therapy has shown promise in improving memory function in post-COVID-19 patients with cognitive impairments.^76^

**Personalized Risk Assessment:** Developing tools for personalized risk assessment could help identify AD and PD patients at the highest risk for severe long-term neurological consequences following COVID-19. This may involve genetic profiling, baseline clinical characteristics, or biomarker patterns.

**Impact of COVID-19 Variants and Vaccination:** Assessment of the impact of different COVID-19 variants and vaccination status on long-term neurological outcomes in AD and PD populations is crucial. This information can guide public health strategies and individualized patient management.

**Health Services Research:** Studies evaluating the effectiveness of different management strategies and care models for AD and PD patients with COVID-19 history are needed to optimize clinical practice and healthcare resource allocation.

## Conclusion

The long-term neurological consequences of COVID-19 in patients with pre-existing Alzheimer’s disease and Parkinson’s disease are significant and multifaceted. Evidence suggests that these patients may experience accelerated disease progression, exacerbation of both cognitive and motor symptoms, and worsened overall outcomes following SARS-CoV-2 infection. The complex interplay between COVID-19 and neurodegenerative processes involves multiple mechanisms, including neuroinflammation, oxidative stress, vascular damage, and potentially direct viral effects on vulnerable neuronal populations. These mechanisms may synergistically accelerate the underlying neurodegenerative processes, leading to more rapid cognitive decline in AD and worsened motor and non-motor symptoms in PD. However, it is possible that factors such as reduced social interaction during the pandemic contributed to observed cognitive declines. Thus managing these patients presents unique challenges, requiring close monitoring, personalized treatment adjustments, and comprehensive support for both patients and caregivers. Emerging evidence suggests potential benefits of early intervention, targeted rehabilitation strategies, and neuroprotective approaches, though further research is needed to optimize these interventions. As our understanding of these complex interactions continues to evolve, it becomes increasingly crucial to develop targeted strategies to mitigate the long-term neurological impact of COVID-19 in these vulnerable patient groups. The COVID-19 pandemic has highlighted the intricate connections between systemic health, infectious diseases, and neurodegenerative processes. As we move forward, integrating this knowledge into our approach to AD and PD management will be crucial in providing optimal care for these vulnerable patient populations. Future research focusing on long-term longitudinal studies, mechanistic investigations, biomarker development, and targeted therapeutic interventions will be essential in improving outcomes for AD and PD patients in the context of the ongoing COVID-19 pandemic and its aftermath.
